# From translocalization to commuterization: Chinese migrant factory workers’ changing arrangements of welfare and care

**DOI:** 10.1007/s10624-025-09769-9

**Published:** 2025-03-27

**Authors:** Yueran Tian

**Affiliations:** Faculty of Sociology, Bielefeld University, Bielefeld, Germany

**Keywords:** Social reproduction, Labor mobility, Urbanization, Housing debts, Welfare, Care

## Abstract

Based on a 10-month fieldwork in central China and 60 in-depth interviews, I explore in this paper a paradoxical phenomenon where the expansion of institutional provision of welfare fails to reach its goal of safeguarding people’s well-being but intensifies the condition of insecurity experienced by migrant factory workers and their families. Recent developments in metropolitanizing spaces through land-for-welfare policies, in-migration of capital, and industrial rezoning have incorporated those at the urban fringes into the Chinese state’s regional development plan. Due to land expropriation, villagers living at the urban edges are gradually replacing the rural land with cash compensation, resettlement housing, residence-based social insurances, and factory employment to secure their livelihoods and maintain social relations. As the factory is located in the neighborhood, former peasants’ trajectories of travel oscillate more frequently between work and home. This creates a new mix of care wherein thin social protection provided by the state allows deeper penetration of market actors. In response to the changing welfare landscape, peasants-turned-urbanites constantly have to make trade-offs between hypermobility and stability: they follow the seasonal hiring rhythm of the factory that provides flexible wages and discourages welfare participation; or they adhere to the rules of the welfare system which requires long-term stay in the factory with low pay. As a result, workers and their families either rely on unstable income to secure their future or participate in social protection schemes in combination with speculative financial activities such as utilizing the Housing Provident Fund together with reduced-interest mortgages. People’s response to the deepening commodification of land, labor, and money therefore emerges from their everyday practices and arrangement of care. In other words, how people react to the conditions of insecurity remain entangled with the welfare structures that create them.

## Introduction

On April 11, 2022, an X-Smart factory worker for 10 years, Xiaozhuang,^1^ invited me for a quick chat after 19:00 because his overtime hours had been canceled for the day. It was a rare opportunity for me to talk to workers on a weekday evening, since they are usually either on night shifts (20:00–8:00) or too tired to interact with others after finishing the day shift (8:00–20:00). Each rotation can last up to twelve hours, including eight regular working hours, a 1-h break, and a maximum of 3 h of overtime. Exhausted after work, workers normally spend 2 to 3 h cooking and eating and then go to bed so that they can have enough sleep before the next shift starts. Faced with only a small window of opportunity, I visited Xiaozhuang and his wife Xiaoyun, who rented a room in the most populated urban villages (*chengzhongcun*) within walking distance of the factory, where trans-local migrant workers like them look for cheap accommodation, food, clothing, and other daily essentials (Chu et al. 2022; Zhan 2015, 2018).

X-Smart is located in New Town, a newly established special economic zone (SEZ) in China’s central inland region. For the past 15 years or so, New Town and its surrounding area have undergone dramatic changes. As the state-led land expropriation was taking place, some local peasants were relocated to high-rise apartment buildings while many were waiting in temporary housing projects and relying on income from a combination of rent, subsistence farming, and gig work. Xiaozhuang and his wife, Xiaoyun, who come from a rural village 30 min by car from New Town, chose to rent a room instead of paying 150 yuan per month per person to stay in the factory dormitory. Their landlord was a New Town native and his family received four compensated apartments due to the loss of rural land. He leased one of them to factory workers and street vendors. In this 60-m^2^ apartment, the landlord had turned both the kitchen and the living room into three 15-m^2^ units for rent in addition to the two small bedrooms. The couple’s room costs 350 yuan a month plus 60 yuan or so for electricity and water. Although it is 110 yuan more than dormitory rooms, this type of housing offers privacy for couples and a good night’s sleep without eight dorm mates working different shifts coming in and out.

Xiaozhuang started his employment with X-Smart in Shenzhen, one of the most densely populated cities in the Pearl River Delta, but in 2011, after marrying Xiaoyun, he decided to move inland with the factory. Xiaoyun had stayed in the rural village with their two children for a couple of years before deciding to take a job at X-Smart and live with her husband to improve the family income. Their children stayed in the rural home and received care mostly from their grandparents whose livelihoods relied on subsistence farming. “I wouldn’t have come out here if his wages were high,” said Xiaoyun. Xiaozhuang admitted that they had to leave the children in the village because their combined income was not enough to support four people living in the city, especially as his monthly wages (4000–5000 yuan) could barely cover the repayments for his car loan and housing mortgages, which amounted to 5000 to 6000 yuan per month. While half of their income was used to cover debts, the other half, namely most of Xiaoyun’s wages (3000–4000 yuan), covered the couple’s daily expenses in the city, their children’s school fees in the countryside, and the costs of gas and gifts for frequent home visits.

Like many trans-local migrants who live and work across rural and urban areas, Xiaozhuang and Xiaoyun left their children in the countryside, where all family members had their *hukou* or “household registration.” Resembling an internal passport system, the *hukou* system divides the Chinese population along rural–urban lines, tracks individual’s mobility trajectories, and restricts those who have rural household registration*.* This includes most migrant workers from the countryside and affects their ability to access urban social welfare and services. At the same time, the *hukou* system grants them access to collectively owned land and limited welfare in their rural area of origin (Andreas and Zhan 2016; Chuang 2020; Chuang and Yasuda 2021; Pan 2018). In this institutional setting, cheap and crowded dormitories house large number of workers and reduce the everyday living costs which should be covered by workers’ wages (Pun and Chan 2013; Smith and Pun 2006). Furthermore, factories in the city avoid the costs of migrant household reproduction, as the rural families and the local state where they live bear these costs through subsistence farming, self-built housing, cheap schooling, and limited pension and health insurance coverage (Chuang 2020; Pan 2018; Zhan 2019). These social reproductive arrangements have sustained capital expansion—particularly along China’s coastal areas—while putting workers and their families in precarious conditions where their wages are low and the welfare provision is thin (Chuang 2015; Fan 2007; Gaetano and Jacka 2004; Lin and Nguyen 2021).

If we zoom in more, the couple’s story further suggests that these precarious conditions are shaped by and shaping the gendered and generational arrangements of household reproduction. Xiaoyun, the wife of Xiaozhuang, and her parents-in-laws took care of the youngest generation of the household as Xiaozhuang migrates to the city and works at X-Smart to subsidize the expenses at home. Similarly, research by Jacka (2010) in rural Shandong, Beijing, and Sichuan demonstrates that women’s labor was essential for child care and subsistence farming. She also points out that intergenerational division of labor intersects with gender where grandmothers in particular took on the responsibility of caring for their grandchildren, who could not live in the city with their migrant parents due to a lack of access to education. Compared to commodified labor, which contributes to economic growth, unpaid labor, which is often performed by women and the older generation and is essential for workers’ everyday living and biological reproduction, has been regarded as less valuable. This artificial division between productive and reproductive labor (Gaetano and Jacka 2004; Jacka 2010; Murphy 2002, 2021; Zavoretti 2020) also conceals the state’s agenda to transfer its responsibility for welfare provision onto the migrant households.

In China, the spatial separation of social reproduction—where the majority of household reproductive tasks are left in the countryside while wage work takes place in urban areas—is far from incidental. Rather, it emerges as a deliberate response to political-economic imperatives and state policies, and as a strategy of migrant families seeking to safeguard their well-being (Chuang 2015, 2020; Jacka 2018; Nguyen and Locke 2014; Nguyen and Wei 2023). By mitigating the reproductive costs of labor, this arrangement effectively subsidizes migrant labor for urban industries, absorbing many of the personal consequences of labor commodification. In doing so, the risks of labor mobility are redistributed across trans-local households, who bear the social and financial burdens necessary to keep the market-socialist system running.

Yet, as Xiaozhuang and Xiaoyun’s story reveals, the once-effective trans-local arrangements can no longer fully cushion migrant families against the harms and risks of labor commodification. While they benefit from more frequent home visits, they remain burdened by the rising costs of household reproduction—particularly in light of the debts they have accumulated. In order to sustain their own livelihoods and to secure a better future for their children, workers feel compelled to meet the factory’s demands for hyper-flexibility (Dong 2022; Feng 2019; Nguyen et al. 2024), where overtime and dispatch work without social protection (which I will discuss in the following sections) align with the factory’s seasonal production schedule and priorities to reduce labor costs. These dilemmas highlight how ongoing welfare restructuring and spatial transformations in China’s inland regions have significantly altered trans-local social reproduction. Against this backdrop, this paper aims to uncover the changing social reproductive patterns and their implications on migrant factory households’ everyday life.

Based on a 10-month ethnographic fieldwork in New Town located in central China and 60 in-depth interviews, I highlight in this paper a process that I refer to as “commuterization,” in which the regional peasantry become incorporated into the industrial labor force as a result of land commodification in China’s central region. In practice, it refers to the daily movements between home and workplace by workers whose family homes are within the vicinity of X-Smart, but more broadly it denotes a spatial transformation that shapes changing patterns of labor mobility and household reproduction. This differs from the trans-local migration that is well documented by the existing literature, which refers to mobility over longer physical distances that do not allow going to work and returning home within the same day and therefore requires a second home around the workplace (Gaetano and Jacka 2004; Jacka 2010, 2018; Nguyen and Locke 2014). That said, industrial relocation from the coast to New Town also shortens the distance of trans-local migration in the region. Unlike workers who travel across regional borders and only return to the countryside once a year for the Spring Festival, trans-local migrant factory workers at X-Smart move within the central region and are able to return home more regularly.

Furthermore, new patterns of household reproduction emerge from the spatial and temporal reorganization of people’s lives in New Town. In the course of the area’s transformation, the commodification of land unleashed by the land restructuring and induced by industrial development is not only uneven in terms of redistributing compensation and land-related welfare, it also generates processes that position people differently in terms of how they can participate in market exchange. That is, while some are able to reap the benefits of the land commodification and put these into profit-making ventures such as housing rentals targeting trans-local migrant workers, others are excluded from this and have to rely on selling their labor and thus turning into commuters. By analyzing how different groups of workers use available forms of welfare for household reproduction, I demonstrate that land commodification makes the reproduction of labor more marketized. In other words, commuterization not only involves changes in the trajectories of workers’ mobility, it also helps induce the privatization of care responsibilities. As a result, it raises workers’ immediate social reproductive costs and increases their greater reliance on cash incomes.

This paper is organized as follows: section “Land restructuring and industrial relocation” sets the context of my study, focusing on X-Smart’s location and the broader land-restructuring and industrial-relocation initiatives in central China that trigger commuterization. Sections “The commodification and disappearing welfare function of rural land” and “Redistributing land compensation and renegotiating care provision in commuting households” investigate how land restructuring in New Town changes people’s arrangement of care, paying attention to the generational and gendered negotiations. By analyzing how the commodification of land intertwines with the commodification of labor, I demonstrate how both factory and household reproductive work become increasingly precarious, thus demanding people to continuously adjust to uncertainties. In section “Trans-local migrants and their new arrangements of care,” I return to trans-local migrants’ household reproduction and highlight the changes brought about by commuterization. Specifically, I illustrate how housing mortgages enable workers to meet their household needs by drawing their wages into the circuits of money through credits. Finally, the conclusion summarizes the main findings and demonstrates how my analysis contributes to our understanding of social reproduction, welfare, and care.

### Land restructuring and industrial relocation

Metropolitan Region Z is one of the most popular destinations for migrants in the central and western regions of China. New Town is an emerging urban space located on the rural fringes of Metropolitan Region Z, which has been expanding for the past 15 years. In the first stage of the development, X-Smart was the biggest foreign direct investment (FDI) project in the area. Some of my interlocutors who started working for the factory before 2010 told me that internally the company had encouraged them to move from Shenzhen in the Pearl River Delta back to their hometown. One of the incentives was promotion and higher wages associated with these workers’ elevated ranks. Being able to work closer to home matters significantly to migrant workers. Although wages are higher in coastal cities like Shenzhen, where most export-oriented factories are located, my interlocutors, especially those who are married with children, prefer to stay close to home.

The movement of workers back from coastal areas to their home regions further inland is an incremental change. For decades, China has been known as the world’s factory. Labor-intensive industries were clustered in the Yangtze and Pearl River deltas when transnational corporations such as Nike and Apple subcontracted their product manufacturing to China. Easy access to ports and a lack of regulation gave these areas their advantages when it came to building factories and recruiting cheap labor. This has been gradually changing as the coastal cities seek to produce higher value-added products and to shift to technology-driven sectors. It is becoming costlier for labor-intensive industries to operate in their former coastal locations because of changes in environmental and labor regulations (Yang and Gallagher 2017). More frequent labor protests and the tightening of labor law enforcement also increase wages and strengthen social protection in the coastal provinces, leading to higher labor costs for these industries. Increasing labor costs were the main factor that led X-Smart to plan its relocation as early as 2010, when it opened up several production sites in southwest and central China. Although their sites in Shenzhen still operate, they have become sites where engineers go for training and are therefore situated at the top of the organizational hierarchy.

For the workers, the expansion of city boundaries and the decreasing income gap in factory work between the coastal and inland regions are among the reasons they end up travelling across the country and finding jobs close to home. Inland provinces with large populations that used to be major sites of out-migration gradually managed to encourage their workers to stay. From 2010 to 2020, the inflow of population into Metropolitan Region Z more than tripled, reaching almost four million. In transforming the landscape of land and labor mobility initiated by the local state and accelerated urbanization, the welfare provision for migrant factory workers and their families has also changed, as the next section shows.

### The commodification and disappearing welfare function of rural land

In the urban areas, trans-local migrant workers’ everyday livelihoods depend mainly on their limited wages as well las the factory’s provision of cheap dormitories and canteen food. Previous scholarly work has shown that the dormitory labor regime (Pun and Chan 2013; Smith and Pun 2006) keeps workers living in dormitory complexes within the vicinity of the production sites, thus helping the factory extend its disciplinary measures to the reproductive sphere of the labor force and reduce the costs of maintaining workers’ everyday life. Although workers find the conditions of food and dormitories unsatisfactory, they are able to reduce everyday expenses and save money to remit to their rural homes. In the rural areas, peasant households use land to gain additional financial support through subsistence farming. Combined with household savings, these incomes support the migrant households when migrant members lose the ability to earn wages due to unemployment, sickness, pregnancy, or when their children are too young to enter the labor market. Moreover, rural homestead land also provides housing for the elderly and the young when the middle generation out-migrate to find work. Finally, peasants’ access to other social provisioning, such as education, is conditioned upon their land-use entitlements and rural *hukou.* This allows migrant workers to leave their children in their rural homes, as most migrant children are not eligible for urban schools or face high costs to attend. Previous scholarly works thus suggest that rural land is essential for trans-local householding because it reduces life course risks associated with migrant work and cushions the harm of welfare exclusion that migrants experienced in the city (Chuang 2015, 2020; Gaetano and Jacka 2004; Jacka 2018; Murphy 2002).

However, the social security function of rural land gradually diminishes due to the commodification of land–that is, the process in which the use of land becomes subject to market exchange. This is triggered by the local states’ urbanization and land finance policies. In the earlier stages of land commodification, local states lease or sell repurposed agricultural land to industrial corporations and real-estate companies in exchange for government revenues. In recent years, they also start to gain cheap credits—borrowing from banks at a low interest rate or issuing low-yield government bonds—using land slated to construct public infrastructure such as welfare housing, railways, and parks (Rithmire 2015; Wu et al. 2020). Existing scholarship (Andreas and Zhan 2016; Chuang 2015, 2020; Lee 2016) suggests that the commodification of land allows local states to make profits and underwrite debts, but at the same time undermines rural land’s role in absorbing the harm of capitalist labor value-extraction.

In the New Town area, land restructuring and the in-migration of capital bring the site of the renewal of migrant labor force closer to that of maintenance (Burawoy 1976). By examining migrant labor systems in southern Africa and the USA, Burawoy (1976) argues that the separation is institutionalized through statecraft so that migrant workers become dependent on their employers for their everyday livelihoods and their home place and family labor for renewal. In similar ways, Chinese factory workers’ day-to-day livelihoods and family life are kept separate through the *hukou* system: the former takes place in the urban dormitories within the industrial compounds, the latter in the countryside (Chuang 2020; Nguyen and Wei 2023). The diminishing spatial separation of migrant labor force reproduction in the New Town area forms the basis for the changes in migration patterns and household reproduction from trans-local to commuting.

In the next section, I tell the stories of two commuters, Sister Feng and Zhang Wen, and their households, whose lands have been expropriated by the local state and whose members have become involved in factory work. Through their cases, I demonstrate that the reconfigurations of labor and welfare are shaping and shaped by the generational and gendered negotiations of care, including the redistribution of land compensation and the arrangements of child care within factory workers’ households. As New Town residents become embedded in the uneven processes of land commodification, how they respond to the increasing insecurities brought by land loss depends on their positioning within these negotiations.

### Redistributing land compensation and renegotiating care provision in commuting households

Sister Feng and her family had lived on a temporary relocation site for more than 3 years when I first met them in January 2021. More than a thousand people from Sister Feng’s village who had once lived in a concentrated location were separated into four sites waiting for their resettlement apartments. Sister Feng told me that in a year or so they would be able to move into the high-rise apartment building not far from their old village. By then each household member would be entitled to 60 m^2^ of living space, and the whole household would get one 60-m^2^ apartment and three 120-m^2^ apartments. Sister Feng and her husband decided to take the small apartment and reserve the three big ones for her grandson, teenage son, and the older son’s household, including his wife and daughter. Although in the official documents, each member of the household, despite gender and age, is entitled to equal compensation, the arrangement of relocation apartments still follows the patriline and shows the pattern of descending familism (Yan 2016, 2021), that is, the youngest generation in the family now receives the most attention through material support, in contrast to the filiality-based notion that the elders should receive the highest privileges.

Sister Feng’s village lost their land bit by bit. First, the residents were moved out of their houses and subsequently lost their homestead land because the local government planned to merge several neighboring villages as part of an urbanization project called “shanty-town renovation” (*penghuqugaizao*). By the time of my fieldwork, their farming land had started to shrink from about 1.3 *mu* to less than 1 *mu* per person, and it was rezoned for a public park project. Although Sister Feng’s household had about four *mu* of land left, they could not rely on subsistence farming alone for their everyday livelihoods. Between subsistence farming and factory work, Sister Feng preferred the latter. Recalling her experience at X-Smart, she told me that she liked the routine:

Factory work is much easier than working in the fields. I worked on the assembly line. You have air-conditioning in the workshop, so you don’t feel the seasons at all. I live close to the factory, so I didn’t have to pay for accommodation. I always worked overtime, so the wage was pretty good. Normally I went to work at six, six thirty. I don’t like to be late. Then I came back around eight thirty or nine (at night). It’s good money. As a permanent worker, you get four thousand something every month.

Sister Feng found the factory job attractive when she could supplement it with subsistence farming and had her family house in place. She and her family had shared about five *mu* of agricultural land, where she could grow her own vegetables and wheat for family consumption. However, after four consecutive years, Sister Feng quit her factory job and became a full-time stay-at-home grandmother. While chatting with me, she picked up her screaming grandson and looked slightly overwhelmed: “He will be three in two months. I will go back to the factory after sending him to the kindergarten. Now both of his parents are working at the factory and can’t take care of him.” The 28-year-old father, Sister Feng’s older son, had been unemployed for a year before he joined X-Smart as a dispatch worker, who only worked for 1 to 3 months during the factory’s peak season of production. After giving birth to two children, his wife also went back to work at the factory on a seasonal basis. Unlike permanent workers, dispatch workers do not receive any welfare benefits but a lump-sum payment about 7000 to 10,000 yuan at the end of their contracts, in addition to the monthly basic wages ranging from 1900 to 2300 yuan. Gesturing toward the room with a closed door, Sister Feng told me that in there was her 16-year-old son who had dropped out of high school and stayed home for more than a year. The third time I saw Sister Feng in May 2021, she still had not gone back to work. She said: “Now my granddaughter goes to school a bit further away from our home. Actually, it is close to her maternal grandparents, but they don’t want to pick her up. The grandpa is a security guard, and it seems like the grandma is working in the factory.” With multiple households in her extended family involved in factory work, Sister Feng compromised and took the responsibility of care.

In rural Guangzhou, located in the Pearl River Delta, Santos (2017) explains why the middle generations take the support of their seniors for granted. He found that migrant parents show their care for their children by prioritizing their “breadwinner” duties over their “nurturant” roles, assuming that there are enough resources in the village for their children. Yet, as Santos’ senior interlocutors explained, taking care of the grandchildren needed the coordination of multiple households (Santos and Harrell 2017, pp. 101–102). In transforming spaces like the New Town area, Sister Feng could no longer rely on agricultural work, nor does she receive direct help from other household members. Furthermore, the cash compensation that the family received of around 300,000 yuan mostly went to Sister Feng’s two sons: covering their expenses during the unemployment period and paying for the older son’s car. Without her factory job, meanwhile, Sister Feng mainly relied on her husband, who worked intermittently as a construction worker. Dissolving collective life due to the land transformation and the slow shift to industrial work therefore continue to put a burden on the grandmother and intensify intergenerational exploitation.

An opposite configuration of family relations is also taking place: the senior generation refuses to take care of their grandchildren, and their daughters-in-law become the sole caretakers of the household. I met Zhang Wen in March 2021 after she had just completed a temporary contract and was waiting for the seasonal bonuses of about 10,000 yuan to appear in her account. Zhang Wen was 35 years old and had been married for more than 10 years. Before relocating to the new apartment, she had lived together with other members of the family under one roof, including her two in-laws, two children, and her husband. At the beginning of Wen’s marriage, she was able to work and took assembly-line jobs in the coastal area. Wen told me that, shortly after returning from Shenzhen, her sister-in-law, who was also her co-worker, filed for divorce. The family later found out that she had run away with the line manager whom she had met at the factory. Zhang Wen took all the blame since her in-laws made her promise to “take care of each other” when going out of town for work. She later realized that this meant that she and her sister-in-law should watch out for potential affairs, as the in-laws did not trust women like them who joined the family from another distant village through an arranged marriage. Ever since the incident, Wen stopped working, and her mother-in-law insisted that she should stay home to take care of her two children.

It was a relief for Zhang Wen when the relocation took place since her own household, including her husband and two children, could move out of the big house and live alone in an apartment. She could then avoid the constant supervision of her mother-in-law and having to do chores for the whole family in the big house. The housing area for relocation apartments is re-distributed within the family based on the number of male-headed households. For purposes of cash compensation, Zhang Wen’s father-in-law was the one who received the money from the local government, while she was kept in the dark when it came to redistribution. Wen’s mother-in-law used some of the compensation to run a small grocery store close to the factory, claiming that she needed to work every day and could not help Wen take care of her children. Wen’s husband not only refused to give her a share but also used all the money for gambling. He had more than a million in debts, due to which he was in prison for some time and had to rely on Zhang Wen and his parents to bail him out. In addition to formal punishment, gambling away essential yet limited resources crucial for the family’s future well-being has been met with strong moral condemnation in Zhang Wen’s village. Similarly, in many migrant-sending villages across rural China, Steinmüller (2011) finds that gambling becomes socially unacceptable when the financial risks threaten a person’s ability to meet certain moral expectations, for instance, of fulfilling a son’s duty to care for his parents and a husband’s role in supporting his nuclear family. In the case of New Town residents, they acknowledge the high uncertainty associated with land loss and are unwilling to take on additional risks through gambling. However, for Zhang Wen, she had to bear the consequences of both. As a result, she could neither benefit financially from the land compensation nor count on her husband or in-laws to attend to her children’s needs.

In order to earn a living and care for her children simultaneously, Zhang Wen began to work for X-Smart. However, she could only work at the factory when her children were in school because during the semester breaks she had to be home cooking or taking her children to different extracurricular classes. Therefore, every time X-Smart started to recruit seasonal workers, Wen would work there for couple of months, save some money, and mostly spend it on both her children’s tuition and everyday expenses for couple of months. She would then return to X-Smart when the money ran out. A close look at intergenerational relations shows that compensation for the loss of land does not provide “freedom” for which Wen had hoped. The gendered hierarchies embedded in Zhang Wen’s family life are shaped by the patrilineal kinship structure but also are deeply rooted in the division between *nei* (inside) and *wai* (outside) (Jacka 2010). While factory and migrant work is considered as *wai*, child rearing and supporting one’s husband is considered as *nei* and as women’s work (see also Chuang 2016). Additionally, Zhang Wen has been treated as an outsider who married into her husband’s family and thus received little trust. As the senior generation controls the land compensation and the uncertainties of land loss rise, she is expected to fulfill both roles of the care taker and the breadwinner. This is when factory work enters into her arsenal of improvisation. Yet, the work, better paid but stripped of any kind of social protection, just barely covers immediate household reproductive needs.

Both cases of Sister Feng and Zhang Wen demonstrate that the social reproduction of commuter households is reorganized in the context of land compensation and a gendered and intergenerational morality. Despite the cash and housing compensation, options like household care and factory jobs, which individuals resort to maintain their household reproduction after losing their rural land, are growing increasingly uncertain. What I found in my field thus is similar to what Tania Li (2020) finds in her later work on the land dispossession in highland Indonesia, where the commodification of rural land subjects household economies and care systems to the logics of the market, drawing differently positioned actors to participate in this process while restructuring previously existing unequal relations. In the context of central China, landless peasants are incorporated into the state-initiated accumulation processes and local welfare system through the land-for-welfare schemes (Cai 2016; Cai et al. 2020; Zhan 2019; Zhan 2021) during which the gendered and generational hierarchies embedded in workers’ household relations are reconfigured. The unevenness of land commodification gives rise to what Dong (2022) calls “hegemonic precarity,” where the increasing care needs within workers’ households require them to seek flexible solutions, which, in turn, feeds into the gig manufacturing regime. In other words, although the precarious work of care (Gaetano and Jacka 2004; Jacka 2010, 2018; Murphy 2002) is as important for commuterization as it is for translocalization, the commodification of rural land and the precaritization of factory work further intensify the mosaic arrangement of care for commuters.

### Trans-local migrants and their new arrangements of care

In this section, I revisit the dynamics of trans-local migrants’ household reproduction and address the puzzle posed at the beginning of this paper: How do Xiaozhuang and Xiaoyun become incorporated into X-Smart’s workforce as a result of commuterization? How does the trans-local arrangement of care by workers like Xiaozhuang and Xiaoyun differ from that of migrants who live and work across regional borders? To explore these issues, I first return to their story, where Xiaozhuang explained how and why he has accumulated debts over the years and continued to work for X-Smart.

Recalling Xiaozhuang, an X-Smart worker of 10 years, who has to pay off a car loan and a housing mortgage, he earns about 4000–5000 yuan per month. These wages include his basic wage of 2300 yuan and earnings from overtime work. All of his wages are used to pay back his debts while his wives’ wages cover other household expenses. For the couple, a car is important because it allows them to drive home every weekend to spend time with their children and family members, but the frequent visits can also be costly, as they spend more money on gas and gifts. Furthermore, Xiaozhuang and his wife had purchased an off-plan apartment at the county seat^2^ close to their home village. Although the down payment on their apartment nearly exhausted the couple’s 10 years of savings, Xiaozhuang believed that this was necessary because his children would need to go there for secondary education from grades 7 to 12, as there was no middle school or high school in the village. While the children’s grandparents could provide them with accommodation and feed them, the daily commute to the school would have taken at least an hour by scooter. Xiaoyun, Xiaozhuang’s wife, considered this unsafe and was worried that her children would not have enough time to finish their homework and sleep. Finally, as Xiaozhuang told me, “You must work if you want to guarantee a good life for the next generation! (*niyao xiayidai guodehao jiu bixu de gan*).” Continuing to labor thus also means securing well-being for the future. Debts and wages therefore helped to meet the couple’s and their families’ everyday needs and long-term goals.

The close coupling between county-level education and real estate further raises the social reproductive costs of trans-local migrant workers who are now travelling more frequently to their rural homes and hope to provide better education and living situation for their children (Liu 2023; Murphy 2020). Liu (2023) finds that in rural Anhui Province, which is also located in central China, the mounting household debts associated with housing compelled his interlocutors to borrow from others. In the case of X-Smart workers and their families, one of the ways to cover their housing debts is to save from their limited wages while borrowing at a low interest rate through the Housing Provident Fund scheme. Designed to make housing loans more affordable with lower interest-rates than commercial mortgages, the Housing Provident Fund is an instrument created by the state to give formally employed workers some support in terms of getting a foothold on the financialized housing market. As a mandatory savings scheme for permanent contract holders, both X-Smart and workers contribute 5% of the base salary into the fund. To qualify for a housing mortgage, one must have at least a 6-month record of shared contributions between the employer and the employee. The entry point into the Housing Provident Fund therefore sets the first barrier for migrant workers: many would not be able to meet the requirements due to their highly flexible and informal employment. For these reasons, my interlocutors who have access to and used the Housing Provident Fund to purchase apartments are often long-term employees at X-Smart.

Xiaozhuang and Xiaoyun’s story further suggests that the couple have to work around their debt repayments along with arranging different household savings, spending, future care needs, and their employment choices. Housing debt around 3500 yuan per month takes 38% of Xiaozhuang and Xiaoyun’s household income. The vignette at the beginning of this paper also details that in addition to housing debt, Xiaozhuang needs to pay back his car loan as a car is essential for the couple to visit their families more frequently in a flexible manner. If they are scheduled to work overtime on the weekends, they do not have to wait for the bus or pay for car sharing services to go home. They could finish their morning shifts at 8 a.m. and drive directly home from the factory, saving almost an entire day to spend with their children. Dong (2022) finds that in Henan Province, Foxconn mothers need to negotiate between their care needs and the factory production regime, which relies on overtime work and a tired wage scheme to reward and discipline workers who meet production demands. Similarly, Xiaozhuang and Xiaoyun’s case shows that, by prioritizing their children’s education and future well-being, both of them had to keep working at X-Smart for higher wages and to gain access to cheaper credits.

## Conclusion

This paper depicts the process I call “commuterization” in which the regional peasantry becomes incorporated into the industrial labor force in central China. Triggered by the in-migration of capital and state-led land-restructuring, translocalization as both a pattern of labor mobility and a form of household reproduction gradually changes into commuterization. Although the distance of travelling from home to work is now less, workers and their families find the burden of household care has increased. By describing how factory workers and their families strategize and provide care for one another during the course of New Town’s transformation, my ethnography highlights the uneven nature of land commodification and the varying degrees of participation among people in this process. As a result of commuterization, factory workers facing insecure employment and rising debts respond with strategies that further intensify the privatization of care. This, in turn, feeds back into land and labor commodification processes.

By mapping the dynamics of social reproduction, this paper foregrounds ruptures and changes as well as contradictions within the sphere of social reproduction. Informed by a feminist social reproduction framework (Bhattacharya and Vogel 2017; Fraser 2016), and anthropological insights on care (Locke 2017; Nguyen and Locke 2014; Nguyen et al. 2017; Thelen 2015; Weiss 2021, 2022), I approach social reproduction as intersecting processes that (1) condition the reproduction of the Chinese market socialist system, (2) regenerate individuals on a daily and generational basis, and (3) spark broader moral and power struggles over the question of needs and shared responsibilities. My approach helps illustrate how evolving welfare policies and land commodification in central China intersect with migrant factory household’s decisions around employment, mobility, child rearing, and housing, thus complicating the dichotomy between the private and the public, between morality and politics, as well as between everyday ethics and institutional logics (Nguyen et al. 2017; Tronto 1993). Within this field of contestations and negotiations over the care of labor, new forms of social organization emerge (Thelen 2015). One such configuration is commuterization, in which migrant factory workers travel shorter distances but face an intensified marketization of care.

## Footnotes

1 All names used in this paper are pseudonyms.

2 The Chinese administrative division has five levels: provincial, prefectural, county, township and basic-level autonomy. In this hierarchical structure, the county level comes in between the prefectural level (city) and the basic-level autonomy (village). The county seat is normally the urban center of a largely rural area. Xiaozhuang and Xiaoyun’s home village, although close to the factory and New Town, belongs to a different administrative area.
